# Plasma Lipidomic Analyses in Cohorts With mTBI and/or PTSD Reveal Lipids Differentially Associated With Diagnosis and *APOE* ε4 Carrier Status

**DOI:** 10.3389/fphys.2020.00012

**Published:** 2020-01-31

**Authors:** Claire J. C. Huguenard, Adam Cseresznye, James E. Evans, Sarah Oberlin, Heather Langlois, Scott Ferguson, Teresa Darcey, Aurore Nkiliza, Michael Dretsch, Michael Mullan, Fiona Crawford, Laila Abdullah

**Affiliations:** ^1^The Roskamp Institute, Sarasota, FL, United States; ^2^School of Life, Health and Chemical Sciences, The Open University, Milton Keynes, United Kingdom; ^3^James A. Haley Veterans’ Hospital, Tampa, FL, United States; ^4^US Army Medical Research Directorate-West, Walter Reed Army Institute of Research, Joint Base Lewis-McChord, Tacoma, WA, United States; ^5^U.S. Army Aeromedical Research Laboratory, Fort Rucker, AL, United States

**Keywords:** traumatic brain injury, post-traumatic stress disorder, biomarkers, lipids, apolipoprotein ε4, mass spectrometry

## Abstract

The differential diagnosis between mild Traumatic Brain Injury (mTBI) sequelae and Post-Traumatic Stress Disorder (PTSD) is challenging due to their symptomatic overlap and co-morbidity. As such, there is a need to develop biomarkers which can help with differential diagnosis of these two conditions. Studies from our group and others suggest that blood and brain lipids are chronically altered in both mTBI and PTSD. Therefore, examining blood lipids presents a minimally invasive and cost-effective approach to identify promising biomarkers of these conditions. Using liquid chromatography-mass spectrometry (LC-MS) we examined hundreds of lipid species in the blood of healthy active duty soldiers (*n* = 52) and soldiers with mTBI (*n* = 21), PTSD (*n* = 34) as well as co-morbid mTBI and PTSD (*n* = 13) to test whether lipid levels were differentially altered with each. We also examined if the apolipoprotein E (*APOE*) ε4 allele can affect the association between diagnosis and peripheral lipid levels in this cohort. We show that several lipid classes are altered with diagnosis and that there is an interaction between diagnosis and the ε4 carrier status on these lipids. Indeed, total lipid levels as well as both the degree of unsaturation and chain lengths are differentially altered with diagnosis and ε4 status, specifically long chain unsaturated triglycerides (TG) and both saturated and mono-unsaturated diglycerides (DG). Additionally, an examination of lipid species reveals distinct profiles in each diagnostic group stratified by ε4 status, mainly in TG, saturated DG species and polyunsaturated phosphatidylserines. In summary, we show that peripheral lipids are promising biomarker candidates to assist with the differential diagnosis of mTBI and PTSD. Further, ε4 carrier status alone and in interaction with diagnosis has a strong influence on peripheral lipid levels. Therefore, examining ε4 status along with peripheral lipid levels could help with differential diagnosis of mTBI and PTSD.

## Introduction

Mild traumatic brain injury (mTBI) and post-traumatic stress disorder (PTSD) are considered signature injuries among United States soldiers returning from recent conflicts in Iraq and Afghanistan (Tanielian and Jaycox, 2008; Carlson et al., 2013). In fact, nearly 20% of soldiers report sustaining a TBI, whilst 5–15% report having PTSD (Tanielian and Jaycox, 2008). Currently, mTBI is undetectable using conventional neuroimaging techniques in clinical settings (Dretsch, 2010). As a consequence, there is reliance on self-report of injury characteristics and symptoms (e.g., alteration in consciousness), and cognitive disturbances, in order to diagnose mTBI (Kim et al., 2017; Dretsch et al., 2019). Diagnosing PTSD also requires self-reporting of symptoms in order to detect emotional, behavioral, and cognitive problems which emerge in patients after traumatic events (American Psychiatric Association, 2013). However, there is considerable homogeneity in symptoms reported by both patients with PTSD and those experiencing persistent symptoms after mTBI, making it difficult to accurately diagnose and differentiate these conditions (Dash et al., 2010; Laskowitz and Grant, 2016; National Center for PTSD, 2018). To further complicate matters, mTBI and PTSD are often comorbid in soldiers returning from combat, where severe psychological trauma occurs either within the context of mTBI or emerges as a consequence of stress following trauma (Stein and McAllister, 2009; Bryant, 2011; Dretsch et al., 2016; Stein et al., 2016; Avallone et al., 2018; Vasterling et al., 2018). Hence, differential diagnosis of mTBI and PTSD presents a challenge. Therefore, investigations of blood biomarkers may advance research efforts aimed at better classifying these conditions in order to provide appropriate care, diagnosis and treatments to patients suffering from mTBI and/or PTSD.

Biomarker discovery work in TBI has identified several promising blood protein biomarkers: glial fibrillary acidic protein (GFAP), ubiquitin C-terminal hydrolase-L1, neuron specific enolase and S100β (Wang et al., 2018). While these proteins may be useful biomarkers of acute and severe TBI, their use in reliably detecting milder forms of TBI remains to be fully investigated (Adrian et al., 2016; Joseph et al., 2018). At present, there are no blood biomarkers available for PTSD, and only broad inflammatory markers and cerebrospinal fluid (CSF) cortisone levels have been thoroughly examined (Schmidt et al., 2013; Michopoulos et al., 2015). Additionally, long-term consequences of mTBI and PTSD may predispose these vulnerable patient populations to the risk of developing neurodegenerative diseases later in life (Perry et al., 2016; Wilson et al., 2017; He et al., 2019) and are known to lead to worse outcomes when comorbid with other disorders (Lippa et al., 2015; Ahmadian et al., 2019). In that regard, several studies have focused on examining blood amyloid-β (Aβ), neurofilaments and tau levels in individuals with TBI (Bogoslovsky et al., 2017; Kim et al., 2018). Among these, elevated blood Aβ40 and Aβ42 fragments have been detected acutely and chronically after mTBI (Lejbman et al., 2016; Bogoslovsky et al., 2017). However, blood Aβ levels appear to lack specificity for TBI (Posti et al., 2019). As such, in both mTBI and PTSD, there is a current need for biomarkers to help characterize biological changes differentially associated with each disorder in order to improve clinical detection and monitoring of long-term consequences of mTBI and PTSD.

Blood lipids are emerging as candidate biomarkers of neurological disorders given their important role in the maintenance of brain health through their contributions to metabolic, inflammatory and neuro-signaling processes (Piomelli et al., 2007). Recent studies suggest that blood and brain lipids are chronically disturbed in both mTBI and PTSD (Hauer et al., 2013; Emmerich et al., 2016). Further, we and others have shown that major phospholipid (PL) classes involved in providing both structural and functional support to the brain are chronically altered in both brain and blood of mouse models of severe and mTBI (Abdullah et al., 2014; Ojo et al., 2018; Thau-Zuchman et al., 2019), in the blood of soldiers with mTBI (Emmerich et al., 2016) and in CSF of civilians after severe TBI (Pasvogel et al., 2010). Many of the post-TBI changes in lipids that have been described involve alterations in blood and brain polyunsaturated fatty acids (PUFA)-containing PLs required for maintaining membrane integrity and synaptogenesis (Liu et al., 2015; Emmerich et al., 2017; Knobloch, 2017; Fiandaca et al., 2018). Lipid associated factors such as cholesterol may be peripherally altered in TBI as, under normal physiological conditions, brain cholesterol is compartmentalized and restricted to the brain by the presence of the blood brain barrier (BBB) (Cartocci et al., 2016); however, BBB disturbances after TBI (Sulhan et al., 2018) may alter the brain’s cholesterol content by allowing an exchange between peripheral and CNS cholesterol pools which may in turn adversely affect neuronal function. Studies have shown that CSF cholesterol increases acutely after TBI (Kay et al., 2003). Although cholesterol ester (CE) levels are thought to increase after excitotoxic insults to the brain (Malgrange et al., 2015), whether cholesterol and CE levels are chronically altered after TBI and PTSD remains to be investigated. Additionally, other lipids such as diacylglycerides (DG) and ceramides (Cer) play a known role in inflammation (Reisenberg et al., 2012; Grösch et al., 2018) and may be reflective of ongoing neuroinflammatory processes in TBI and PTSD. As such, blood lipids that are altered in response to neuropathological changes following mTBI and PTSD could be useful biomarkers of these conditions and their evolving pathologies.

Apolipoprotein E (ApoE, encoded by the *APOE* gene) is a constituent of lipoprotein particles responsible for transporting lipids from the bloodstream to various tissues including the brain (Jonas and Phillips, 2008; Tudorache et al., 2017). Among the three major *APOE* polymorphisms, the ε4 allele is known to have impaired lipid transport to the brain and is also associated with worse cognitive outcomes in both TBI and PTSD (Mota et al., 2017). ApoE/lipoprotein complexes facilitate lipid transport to the BBB where lipids are processed and subsequently transported into the brain by fatty acid binding proteins (FABP) as well as by other transporters (Mitchell and Hatch, 2011; Sepe et al., 2018). Given the role of ApoE in brain injury and lipid transport, it is possible that different ApoE isoforms may affect blood lipid levels in interaction with injury.

Based on the known role of lipids in response to injury and the potential interaction with *APOE* genotype, we hypothesized that blood lipid levels would be affected both by diagnosis and the *APOE* ε4 allele. Using LC/MS, we examined several major blood lipid classes in a cross-sectional military cohort of soldiers with a diagnosis of mTBI, PTSD or both, as well as healthy controls. Further, we investigated the protein biomarkers FABP3, GFAP, Aβ38, Aβ40, Aβ42 as well as the ratio of Aβ42 to Aβ40, which has been shown to be altered in TBI (Lejbman et al., 2016), to compare lipid changes to protein biomarkers. This study will help determine whether peripheral lipids may be promising biomarkers to eventually help clinicians with the differential diagnosis and prognosis of mTBI sequelae and PTSD.

## Materials and Methods

### Cohort Characteristics and Measurements

The recruitment details of these military cohorts have been previously described in Emmerich et al. (2016), where basic demographics as well as deployment related history, psychological health questionnaires and neurobehavioral symptoms data were collected from two cohorts of 120 active duty male soldiers, pre-deployment to the Middle East for Operation Iraqi Freedom/Operation Enduring Freedom, who participated on a voluntary basis under IRB approved consent. For the Army, a non-deployable status in relation to a psychiatric condition requires a clinician diagnosis in their medical record. Due to the nature of our study design, we did not scrub medical records of soldiers from the respective brigade to maintain their anonymity. Similar to a psychiatric condition, a non-deployable status in relation to a mTBI requires three or more documented injuries in their medical record. Hence all subjects in this study were deemed medically fit for deployment after physical and psychiatric assessments through deployment medical screening. Our diagnostic categories for participants were determined by screening instruments at pre-deployment. All participants were screened for mild TBI (mTBI) and PTSD using the Defense and Veterans Brain Injury Center Brief Traumatic Brain Injury Screen (BTBIS, Schwab et al., 2006) and the PTSD Checklist Military Version where the PCL-M, with a score ≥ 35 was considered positive in order to provide a provisional diagnosis of PTSD. We chose a cut-score of 35 which is suggested when screening in general population samples that have an estimated prevalence of PTSD below 16%. Diagnosis was then assigned by a trained neuropsychologist. Participants were also screened for both depression and alcohol consumption levels, using the Zung Depression Scale (Zung, 1965) and the Alcohol Use Dependency Identification Test (Lundin et al., 2015), respectively. Additionally, level of anxiety was assessed using the Zung Anxiety Scale (Zung, 1971, 1974) and self-perceived stress level using the Perceived Stress Scale (Cohen et al., 1983). Sleep quality was assessed using the Pittsburgh Sleep Quality Index (Buysse et al., 1989) and daytime sleepiness was assessed using the Epworth Sleep Scale (Johns, 1991). Finally, post-concussive symptoms were assessed using the Neurobehavioral Symptom Inventory (NSI, Cicerone and Kalmar, 1995). The numbers per diagnostic groups were the following: 52 controls, 21 mTBI, 34 PTSD, 13 mTBI + PTSD. Additionally, neurocognitive battery, Central Nervous System – Vital Signs test (CNS-VS, Gualtieri and Johnson, 2006) was administered to participants at the time of sampling, CNS-VS includes multiple subtests to assess verbal memory, information processing speed, complex attention, cognitive flexibility, reaction time, and executive function domains.

Non-fasting blood samples were collected throughout the day at phlebotomy stations by staff blinded to the diagnosis status of the study participants using previously established standard operating procedures. Briefly, blood was drawn into EDTA tubes for preparing plasma and DNA genotyping. Samples were coded at the time of collection and remained coded for all subsequent processing and data generation. For preparing plasma and to remove platelets and other blood cells, whole blood was centrifuged at room temperature on site at high speed of 1380 × *g* for 5 min, following separation the clarified plasma was immediately transferred to a 15 mL conical tube supplemented with a 20× protease inhibitor cocktail (PIC) (Roche) containing 0.5M EDTA to a final concentration of 1X PIC and vortexed. Samples were stored in dry ice before transport to a −80°C medical specimen freezer at the end of each day. Additionally, samples from whole blood aliquots were *APOE* genotyped in our laboratory, by purifying the DNA (Gentra Puregene Blood Kit, Gentra Systems), then using standard polymerase chain reaction as described by Emi et al. (1988) followed by electrophoresis with experimenters blinded to participants’ group membership and other group characteristics (all procedures described in more details in Emmerich et al., 2016). Due to low numbers when stratifying by specific genotype in addition to diagnosis (six possible allele combination for four different diagnostic groups) and since most participants had either an ε3/ε3 or ε3/ε4 genotype, participants were grouped into ε4− and ε4+ groups where the ε4− group was composed of participants who did not have any ApoE ε4 allele and the ε4+ group of participants who had one or two ApoE ε4 alleles. The final numbers per subgroups were; 37 ε4−controls, 15 ε4+controls, 16 ε4−mTBI, 5 ε4+mTBI, 24 ε4−PTSD, 10 ε4+PTSD, 8 ε4−mTBI + PTSD, and 5 ε4+mTBI+PTSD.

### Lipidomics Analysis

Plasma from this cohort was analyzed following a randomized block group design with experimenters performing the sample preparation, lipid extraction, qualitative and quantitative mass spectrometry analyses blinded to participants’ group membership and other group characteristics. The extraction procedure and nano-LC/MS conditions were previously published by our laboratory (Joshi et al., 2018). Plasma (10 μL) was spiked with 5 μl of a mix of SPLASH LipidoMIX stable isotope and Cer/Sph Mixture I (Avanti, Polar Lipids, Inc.) diluted 1:10 in SPLASH internal standard (IS) mix. All solvents were HPLC grade purchased from ThermoFisher Scientific. Samples were extracted using a modified Folch method. Methanol (80 μL) was added to the samples followed by vortexing for 1 min before adding 120 μL of chloroform and vortexing again for 1 min. Samples were then centrifuged at 4°C at 20,000 relative centrifugal force (RCF) for 10 min. The supernatant was then transferred to a low retention Eppendorf microtube, and 40 μL of 0.88% potassium chloride was added. Then samples were vortexed for 1 min. Samples were centrifuged as before, and the lower phase was transferred to another low retention Eppendorf microtube and evaporated by vacuum centrifugation. For the cleanup procedure, non-sterile micro-centrifugal filters (Thermo Scientific) were prepared by applying 200 μL of 1:1 chloroform:methanol to the filters and centrifuging at 4°C, 10,000 RCF for 5 min. The flow-through was discarded, and 1:1 chloroform:methanol was added to the samples which were vortexed and then applied to the filters and centrifuged as before. After, the filters were discarded and the flow-through transferred to auto-sampler vials with inserts and dried under vacuum and re-suspended in 50 μL of 70:30 mobile phase A–B, with mobile phase A comprised of 27% isopropanol, 42% water, 31% acetonitrile, 10 mM ammonium formate with the addition of 0.1% formic Acid. Mobile phase B was comprised of 90% isopropanol, 10% acetonitrile, 10 mM ammonium formate, and 0.1% formic acid.

An Easy-nanoLC 1000 instrument was used in combination with a nanoflex ESI source and a Thermo QE/Orbitrap mass spectrometer. Samples were injected into an Acclaim PepMap 100, 75 μm × 2 cm nanoViper C18, 3 μm, 100Å trapping column and Acclaim PepMap RSLC, 75 μm × 15 cm nanoViper C18, 2 μm, 100Å analytical column for lipid chromatographic separation, running the following gradient at a constant flow rate of 250 nL/min. The starting conditions were 30% B, then from 1 to 50 min program from 50 to 98% B, then switch to 30% B from 50 to 65 min. All samples were run in triplicate in batches of 8 along with a blank and quality control (QC) sample. Full-scan MS data was acquired in both positive and negative ion modes, with a mass range of m/z 130–2,000 in the positive ion mode, m/z 220–2,000 in the negative ion mode, at a resolution of 30,000 for both. The heated capillary was maintained at 200°C, with a spray voltage of 1,500 V. A maximum inject time of 200 ms was used with 13 microscans/acquired scan. Peak areas were integrated using the Tracefinder^TM^ software using a target compound list of lipids of interests containing m/z and retention time for each target lipid and IS for that specific lipid class. For each lipid class, the concentration of lipid species was calculated using the spiked IS corresponding to that class (except for hexosylceramides where the closest eluting IS was used, see Supplementary Table S8), by dividing the target compound area by the IS area and multiplying by the known IS concentration spiked in. Each species in a sample run that had a coefficient of variance (CV) > 25% was excluded from further analysis as considered not to have been measured reliably. Each analytical batch was normalized using its QC ([sample] × [batch QC/normalizing QC]).

### Immunoassays

#### Amyloid-Beta

A V-PLEX Aß Peptide Panel (Meso Scale Discovery) was used to quantify Aβ38, Aβ40 and Aβ42 in the plasma samples. All procedures were performed as per the manufacturer’s instructions with samples run in duplicate. Concentrations were obtained in pg.ml^–1^, with a stated dynamic range of 16.7–8475 pg.ml^–1^ for Aβ38, 9.97–7000 pg.ml^–1^ for Aβ40, and 0.368–1271 pg.ml^–1^ for Aβ42.

#### GFAP

A R-PLEX Human GFAP Antibody Set (Meso Scale Discovery) was used to measure GFAP in the plasma samples. As GFAP was at first not detected in the samples following standard instructions, the assay was repeated with samples run neat (without dilution or addition of assay buffer) and left to incubate overnight at 4°C with the primary antibody. The experiment was otherwise conducted as per the manufacturer’s instructions. Samples were run in duplicate. Concentrations were obtained in pg.ml^–1^, with a stated dynamic range of 63–500,000 pg.ml^–1^.

#### FABP3

A FABP-3 Human ELISA Kit (Invitrogen, Thermo Fisher Scientific) was used to measure FABP3 in the plasma samples. As FABP3 was not detected in all samples following standard instructions, the assay was repeated running the samples neat (without dilution or addition of assay buffer). The experiment was otherwise conducted as per the manufacturer’s instructions. Samples were run in duplicate. Concentrations were obtained in pg.ml^–1^, with a stated dynamic range of 156.3–5000 pg.ml^–1^ and an inter-assay coefficient of variance (CV) of 6.2% and intra-assay CV of 3.9%.

For all immunoassays, duplicates with CV > 25% were removed from further analysis. Data from each immunoassay was analyzed separately in SPSS.

### Statistical Analysis

As these were exploratory studies, *post hoc* power calculations were conducted using the G-power software which showed a greater than 97% power at α = 0.05 for total content of specific lipid class, with the observed effect size *f* = 0.98 for total TG, *f* = 1.7 for total DG, *f* = 1.2 for total Cer, and *f* = 1.02 for HexCer for the *n* per groups utilized in this study. Differences in age, genotype and sex were assessed between diagnostic groups by first running a Brown–Forsythe test to examine the equality of variance between groups as group numbers varied. No significant differences in variance were detected so a One-Way ANOVA was performed on each variable. The Fisher’s exact test was performed on each medication therapeutic category to determine whether the distribution of medication use significantly differed between groups. If this was found to be the case main effect and interaction effect with diagnosis and *APOE* genotype were tested using the Mixed Linear Model (MLM) function. For triglycerides (TG), diglycerides (DG), cholesterol, cholesterol esters (CE), phosphatidylserines (PS), ceramides (Cer), and hexosylceramides (Hexcer) triplicate data was uploaded to SPSS for lipidomic analysis and the mean of duplicate data for the immunoassays. The percentage of included/excluded values of each lipid species was reviewed, and those with < 75% of values included were removed from further analysis, as the sample size was considered too small.

For lipids, totals were calculated by adding up all lipid species belonging to that class. Saturated fatty acids (SFA) were calculated by adding up all lipid species with no double bonds, mono-unsaturated fatty acids (MUFA) by adding up lipid species with one double bond and polyunsaturated fatty acids (PUFA) by adding up lipid species with more than one double bond. For grouping lipids by chain length, a principal component analysis (PCA) was performed on individual species which were grouped by chain length based on their correlation coefficients, as some chain lengths were found to be highly correlated. Normality of the data was assessed by plotting histograms. Non-normally distributed data was normalized using the natural log function. The main effect of diagnosis and *APOE* genotype as well as their interaction effect was tested using the MLM function for triplicate data and general linear model (GLM) function for immunoassay data. Based on these findings, a MLM was run for lipidomic data or One way ANOVA for immunoassay data examining factors previously found to be significant. This was followed by multiple comparisons to test for significant differences between groups using least significant difference (LSD), chosen as a less stringent *post hoc* analysis for these exploratory data. This was done for totals as well as for saturation, chain length groups and lipid species. For the heatmap, fold-changes against ε4−controls were calculated *B/A*. For all analyses, a *p*-value < 0.05 was considered significant to minimize the risk of false positives. For the individual lipid species, as many comparisons were made, a Benjamini–Hochberg (*B–H*) correction was performed to control for false positives, with a false discovery rate *Q* set to 0.1. For *B–H* correction *p*-values were sorted in ascending order and assigned with a rank then each individual *p*-value’s *B–H* critical value was calculated (*i*/*m*)*Q* (with *i* = rank, *m* = total number of tests performed, *Q* = the false discovery rate). The largest *p*-value than its critical *p*-value and all other ranked above were retained as significant (Simes, 1986). All comparisons were made against control, ε4− or ε4+ control groups as stated. GraphPad Prism was used to graph all data. Although statistical analyses were performed on normalized data, raw data were graphed for all immunoassays. As each lipid class was quantified using one IS corresponding to that class, the quantification is relative, and data are presented as percent of control in order to be more informative. However, the raw quantified data is available in Supplementary Tables S3–S8. For DG MUFA as well as CE of chain length 22 few lipid species (*n* = 2) were detected and/or passed quality control, for these the data presented may be more representative of changes in few individual lipids rather than class-wide changes in saturation states or chain lengths (*n* ≥ 4 for all others). The ε4 + mTBI + PTSD group was removed from total Cer after data cleanup as its number was too low (*n* = 1).

## Results

### Basic Demographics, Psychological Health, Neurobehavioral and Cognitive Data of the Study Population

Cohorts characteristics are presented in Supplementary Tables S1, S2. Briefly, there were no significant differences in allelic distribution, age, race, mother’s education, previous number of deployment or paygrade (related to rank) between diagnostic groups with or without ε4 stratification. When examining potential confounding factors for medication use, only anti-depressants were found to be a potential confounder. However, no main effect or interaction with diagnosis was observed for antidepressants on the lipid classes examined (Supplementary Table S1), except for CE where antidepressant use was found to be a potential confounder, however, removing participants on anti-depressants did not alter our results. Therefore, in the absence of any confounding by medication use, analyses were performed with the whole cohort. In Supplementary Table S2 we present psychological health, neurobehavioral data and neurocognitive assessment measures. We observe higher NSI scores in the mTBI, PTSD and mTBI + PTSD group compared to controls, indicating endorsement of more post-concussive symptoms as well as higher severity of these symptoms. We also report both poorer sleep quality, increased daytime sleepiness and higher anxiety in our PTSD diagnosis group compared to controls, our mTBI and mTBI + PTSD groups also reported poorer sleep quality and higher anxiety. We also found a higher prevalence of depression in our PTSD group. In these cohorts we did not find any significant differences between controls and diagnostic groups in alcohol abuse, stress and neurocognitive scores (Supplementary Table S2).

### *APOE* ε4 Influences the Association of Neutral Lipids and Sphingolipids With mTBI and PTSD Diagnosis

**FIGURE 1:**
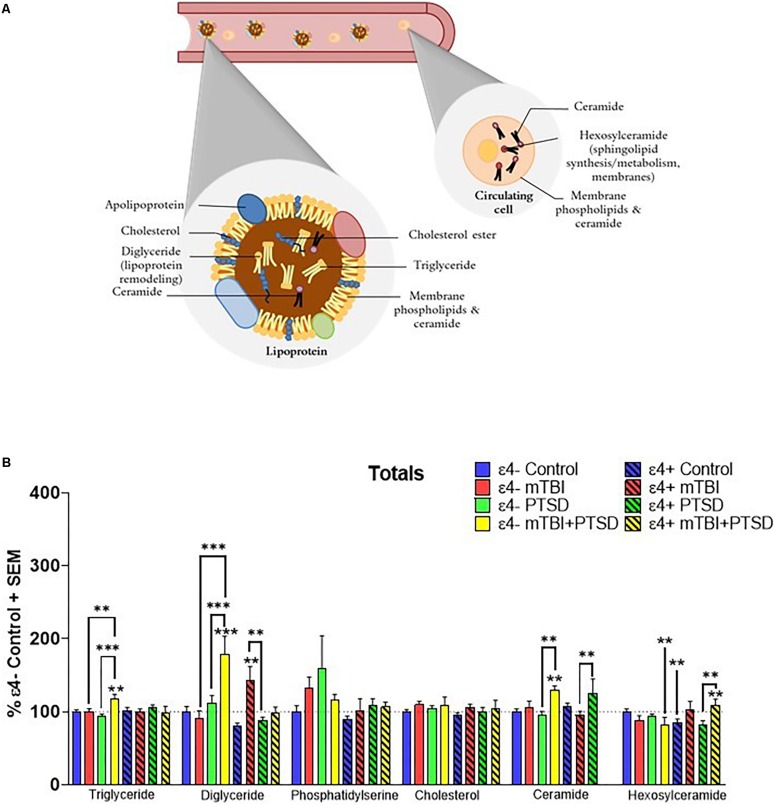
Examination of total peripheral levels of several lipid classes in mTBI, PTSD and mTBI + PTSD in individuals with and without the APOE ε4 allele using an LC-MS assay. **(A)** Schematic illustration of possible main contributors to the signals observed. **(B)** There was an interaction of diagnosis and ε4 status for TG (*p* = 0.029), DG (*p* = 0.000252), Cer (*p* = 0.01), and Hexcer (*p* = 0.003) lipids. Statistical analyses; MLM with LSD *post hoc*, *p* ≤ 0.05 cut-off, ^∗∗^*p* ≤ 0.01, ^∗∗∗^*p* ≤ 0.001. TG, Triglycerides; DG, Diglycerides; Cer: Ceramides; HexCer, Hexosylceramides.

Total plasma levels of TG, DG, CE, cholesterol, Cer, Hexcer, and PS were examined for their association with mTBI, PTSD and mTBI + PTSD diagnoses (Figure 1A). There was an interaction of diagnosis and ε4 status for TG (*F*_(3,345.742)_ = 3.054, *p* = 0.029), DG (*F*_(3,311.703)_ = 6.582, *p* = 0.000252), Cer (*F*_(3,107.561)_ = 3.918, *p* = 0.01) and Hexcer (*F*_(3,243.025)_ = 4.836, *p* = 0.003) lipids. There was a significant main effect of diagnosis and the ε4 carrier status and an interaction between them for total DG levels (*p* ≤ 0.05). There was no effect of diagnosis or the ε4 status on total cholesterol and PS levels. *Post hoc* comparisons of controls showed that Hexcer were significantly decreased among ε4 + controls compared to ε4− controls (*p* ≤ 0.05, Figure 1B). Among ε4− participants, total TG and DG were significantly elevated in the mTBI + PTSD group compared to control and other diagnostic groups (*p* ≤ 0.01, Figure 1B). In ε4+ groups, total DG levels were also increased in mTBI compared to controls and PTSD groups (*p* ≤ 0.01, Figure 1B). Additionally, in ε4− groups total Cer levels were elevated in the mTBI + PTSD compared to controls and PTSD groups (*p* ≤ 0.01, Figure 1B). In ε4+ groups, total Cer levels were elevated in the PTSD group compared to the mTBI group (*p* ≤ 0.01, Figure 1B). Compared to ε4− controls, total Hexcer levels were lower in ε4 + controls and in ε4− mTBI + PTSD participants (*p* ≤ 0.01, Figure 1B). Among ε4 + participants, total Hexcer levels were increased in participants with mTBI + PTSD compared to controls and PTSD (*p* ≤ 0.01, Figure 1B).

### *APOE* ε4 Influences the Association Between the Degree of Unsaturation and Chain Lengths of TG, DG, and CE in mTBI and PTSD Diagnoses

We stratified lipid species by their degree of unsaturation and carbon chain lengths since these parameters relate to metabolic lipid processes and PCA showed high correlation coefficients between chain groups shown here.

**FIGURE 2:**
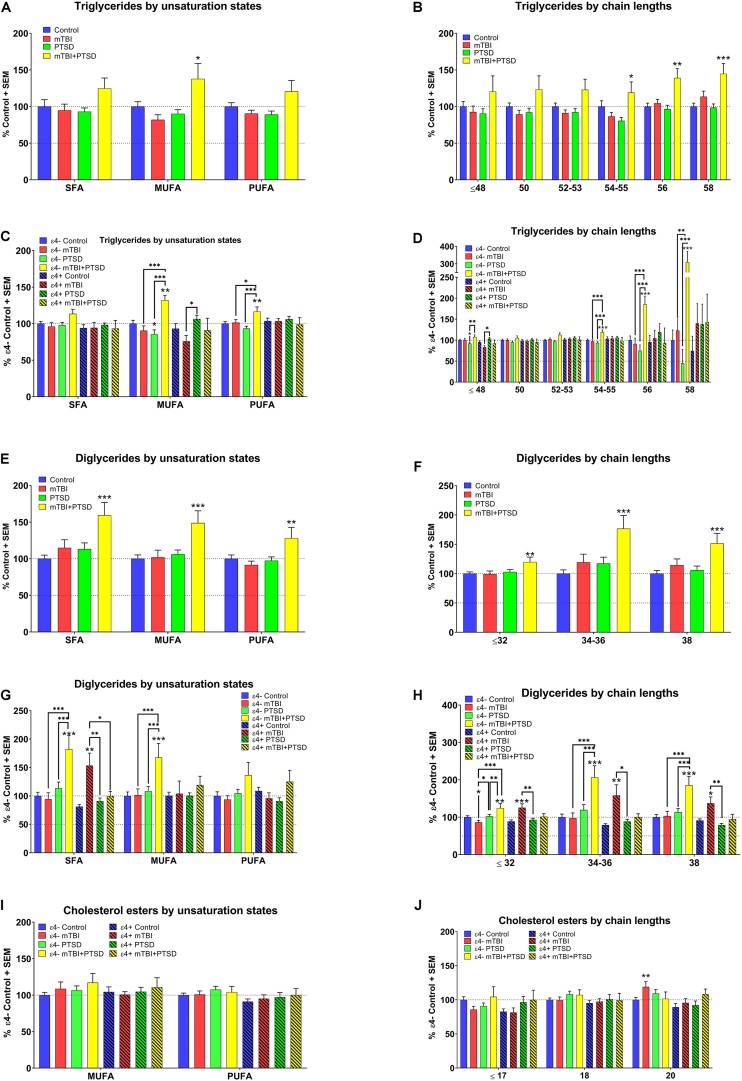
Examination of TG, DG, and CE lipids by unsaturation and chain length status in mTBI, PTSD, and mTBI + PTSD diagnostic groups with and without ε4 stratification. **(A)** There was a main effect of diagnosis for MUFA TG (*p* = 0.054). **(B)** There was a main effect of diagnosis in TG chain length 58 (*p* = 0.026). **(C)** There was an interaction of diagnosis with ε4 status for MUFA (*p* = 0.004) and PUFA (*p* = 0.041). **(D)** There was an interaction of diagnosis with ε4 status in TG chain lengths: ≤ 48 (*p* = 0.007), 54–55 (*p* = 0.011), and 56 (*p* = 0.014). **(E)** There was a main effect of diagnosis in SFA (*p* = 0.001), MUFA (*p* = 0.011), and PUFA DG (*p* = 0.03). **(F)** There was a main effect of diagnosis in DG chain length: ≤ 32 (*p* = 0.035), 34–36 (*p* = 0.001), and 38 (*p* = 0.002). **(G)** There was an interaction of diagnosis with ε4 status in SFA DG (*p* = 0.000208). **(H)** There was an interaction of diagnosis with ε4 status for DG chain length: ≤ 32 (*p* = 0.000415), 34–36 (*p* = 0.001), and 38 (*p* = 0.001). **(I)** No main effect or interaction effect were found significant for CE unsaturation states. **(J)** No main effect or interaction effect were found significant for CE chain length. Statistical analyses; MLM with LSD *post hoc*, *p* ≤ 0.05 cut-off. ^∗^*p* ≤ 0.05, ^∗∗^*p* ≤ 0.01, ^∗∗∗^*p* ≤ 0.001 vs. control (**A,B** and **E,F**), ε4− and ε4+ respective controls (**C,D**, **G,H**, and **I,J**). TG, Triglycerides; DG, Diglycerides; CE, Cholesterol esters; SFA, saturated fatty acid; MUFA, Mono-unsaturated fatty acid; PUFA, Poly unsaturated fatty acid.

A significant main effect of diagnosis was observed for MUFA containing TG species (*F*_(3,351.393)_ = 2.567, *p* = 0.054) with *post hoc* analyses showing that their levels were elevated in mTBI + PTSD participants compared to other groups (*p* ≤ 0.05, Figure 2A). There was an interaction between diagnosis and e4 for MUFA (*F*_(3,351.393)_ = 4.444, *p* = 0.004) and PUFA TG species (*F*_(3,345.366)_ = 2.776, *p* = 0.041). Among ε4− participants, MUFA- and PUFA-containing TG species were elevated in the mTBI + PTSD group compared to other groups (*p* ≤ 0.05, Figure 2C). Compared to ε4− controls, MUFA TG were decreased in ε4− PTSD participants (*p* ≤ 0.05, Figure 2C). Additionally, MUFA TG were decreased in the ε4 + mTBI group compared to the ε4 + PTSD group (*p* ≤ 0.05, Figure 2C). With respect to the examination of chain-lengths in TG species, we observed an effect of diagnosis on TG chain-length 58 (*F*_(3,350.303)_ = 3.136, *p* = 0.026) and interaction between diagnosis and the ε4 carrier status on TG species of TG ≤ 48 (*F*_(3, 351.029)_ = 4.057, *p* = 0.007), 54–55 (*F*_(3,348.046)_ = 3.786, *p* = 0.011), and 56 chain-lengths (*F*_(3,351.984)_ = 3.6, *p* = 0.014). *Post hoc* analyses showed a differential effect of diagnosis for TG ≥ 54 which were elevated in the mTBI + PTSD group (*p* ≤ 0.05, Figure 2B). Additionally, TG ≤ 48 were lower in the ε4− PTSD group compared to ε4− controls and higher in the ε4− mTBI + PTSD group compared to the ε4− PTSD group (*p* ≤ 0.05, Figure 2D). However, TG ≤ 48 were lower in ε4 + mTBI compared to the ε4 + TBI + PTSD group (*p* ≤ 0.05, Figure 2D). Lastly, levels for TG ≥ 54 were elevated in ε4− mTBI + PTSD group compared to all other ε4− diagnostic groups (*p* ≤ 0.01, Figure 2D). For DG, a main effect of diagnosis was observed for all unsaturated species (SFA: *F*_(3,333.756)_ = 6.708, *p* = 0.001, MUFA: *F*_(3,342.936)_ = 3.763, *p* = 0.011), and PUFA: *F*_(3,321.356)_ = 3.017, *p* = 0.03) and chain lengths (≤ 32: *F*_(3,329.790)_ = 2.897, *p* = 0.035, 34–36: *F*_(3,332.523)_ = 5.473, *p* = 0.001, and 38: *F*_(3,330.429)_ = 6.4.893, *p* = 0.002) as well as a main effect of the ε4 carrier status on DG with chain lengths ≥ 34–38 (34–36: *F*_(1,332.523)_ = 4.086, *p* = 0.044 and 38: *F*_(1,330.429)_ = 7.492, *p* = 0.007). *Post hoc* analyses showed that, compared to controls, participants with a diagnosis of mTBI + PTSD had elevated DG levels, irrespective of the degree of unsaturation and chain-length (*p* ≤ 0.01, Figures 2E,F). A significant interaction between diagnosis and ε4 carrier status was detected for SFA-containing DG species (*F*_(3,333.756)_ = 6.708, *p* = 0.000208) and DG of all chain lengths (≤ 32: *F*_(3,329.790)_ = 6.199, *p* = 0.000415, 34–36: *F*_(3,332.523)_ = 5.713, *p* = 0.001, and 38: *F*_(3,330.429)_ = 5.772, *p* = 0.001), where SFA and MUFA DG species were elevated in the ε4−mTBI + PTSD group compared to all other ε4− groups (*p* ≤ 0.001, Figure 2G). In addition, SFA DG were elevated in the ε4 + mTBI group compared to all other ε4 + groups (*p* ≤ 0.05, Figure 2G). Shorter chain length DG species (≤ 32) were decreased in the ε4− mTBI group compared to ε4− controls and the ε4− PTSD group (*p* < 0.05). There was no effect of DG chain lengths on any of the other groups, as their levels were altered in the same way irrespective of DG chain length (Figure 2H).

Among the remaining lipids analyzed, for CE, there was no main effect of diagnosis, only a main effect of the ε4 carrier status for species with a chain length of 20 (*p* ≤ 0.05). There were no differences for the degree of unsaturation (Figure 2I). Cholesterol esters of chain length 20 were significantly elevated in the ε4− mTBI group compared to ε4− controls (*p* ≤ 0.01, Figure 2J). For PS, no main effects or interactions were found to be significant. No significant differences were found in PS levels between groups with or without ε4 stratification (see Supplementary Figure S1).

### Analyses of Lipid Species to Identify Non-overlapping Potential Biomarker Lipids for Each Diagnostic Group

**FIGURE 3:**
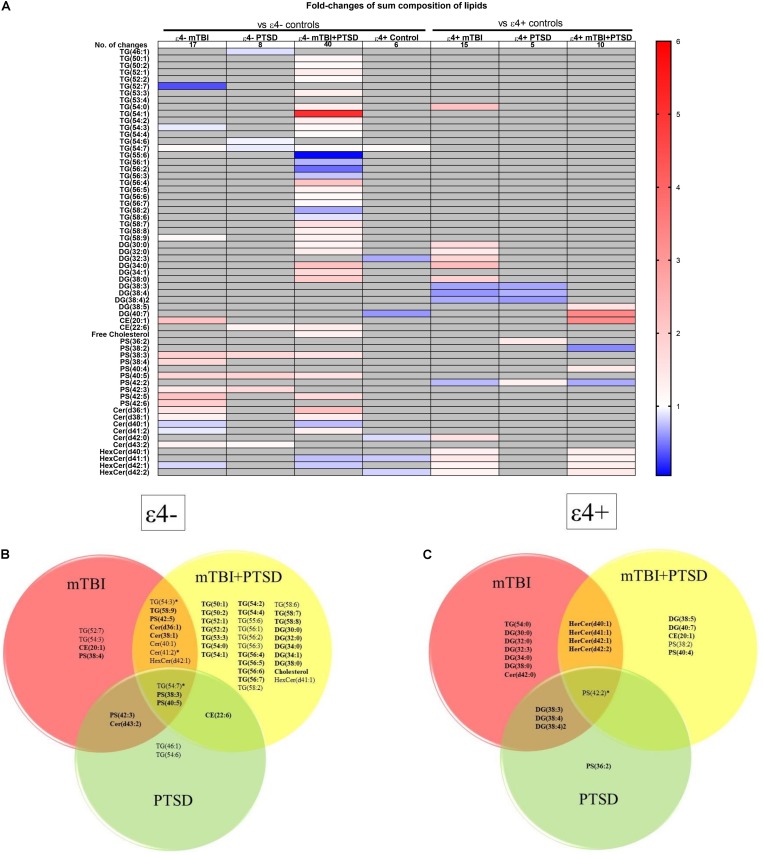
Liquid chromatography-mass spectrometry examination of peripheral levels of sum composition lipids in mTBI, PTSD and mTBI + PTSD in individuals with and without the ε4 allele. **(A)** Heatmap of fold change of lipids that were significantly altered compared to their ε4− and ε4+ respective controls. **(B)** Venn diagram of sum composition lipids altered in ε4− diagnostic groups compared to their control. **(C)** Venn diagram of sum composition lipids altered in ε4+ diagnostic groups compared to their control. Statistical analyses; MLM with LSD *post hoc* and B–H correction, *p* ≤ 0.05 cut-off. TG, Triglycerides; DG, Diglycerides; CE, Cholesterol esters; PS, Phosphatidylserines; Cer, Ceramides; HexCer, Hexosylceramides. DG(34:2)2 indicates a second peak integrated separately for that lipid. In Venn diagrams bolded lipids indicate an increase compared to controls while non-bolded indicates a decrease. Lipids marked by an asterisk indicate this lipid is increased in a group and decreased in another.

We next examined how lipid species profiles differed in mTBI, PTSD and mTBI + PTSD diagnostic groups. There was a main effect of diagnosis and an interaction between diagnosis and the ε4 carrier status for various lipid species shown in Figure 3A. The heatmap (Figure 3A) shows the fold-change of lipids significantly altered in each diagnostic group compared to their respective controls (ε4− or ε4+ controls) in order to examine the directionality of the changes observed in lipid species. The greatest number of changes were seen in the ε4− mTBI + PTSD group with a total of 40 lipids significantly altered compared to ε4− controls, while the ε4 + mTBI + PTSD group had 10 lipids significantly altered compared to ε4+ controls (Figure 3A). Further, both the ε4− mTBI and ε4+mTBI groups exhibited significant changes in over a dozen of the lipid species investigated, each compared to their respective ε4− and ε4+ control groups (Figure 3A). Lastly, compared to other diagnostic groups, both the ε4− and ε4+PTSD exhibited fewer changes in lipid species, with 5 and 10 changes observed, respectively (Figure 3A). Figures 3B,C show lipid species that overlapped between the diagnostic groups. Of note are unsaturated TG, PS and CE species and a number of saturated DG species that significantly differed between mTBI, PTSD and mTBI + PTSD groups in an *APOE* ε4 dependent manner.

### Immunoassay-Based Analyses Detect No Significant Changes in Plasma FABP3, GFAP, and A*β* Levels but a Significant Difference in A*β*42/A*β*40 Ratio Between PTSD and Other Diagnostic Groups

**FIGURE 4:**
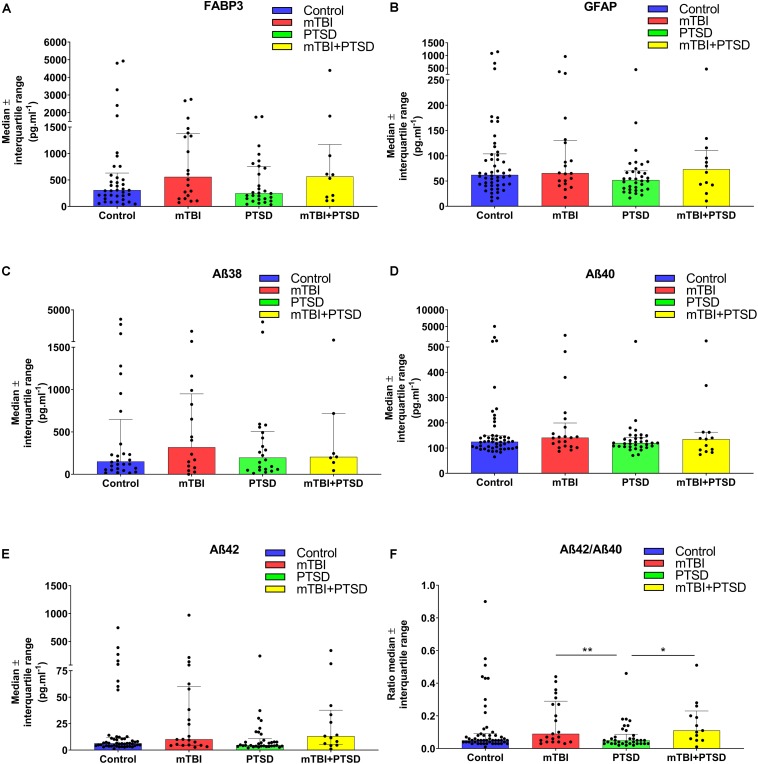
Investigation of plasma protein biomarkers using immunoassays. **(A)** No significant changes were observed between diagnostic groups’ FABP3 peripheral levels. **(B)** No significant differences were observed between diagnostic groups’ GFAP peripheral levels. **(C)** A main effect of diagnosis was observed for Aβ38 (*p* = 0.045) but was non-significant in the ANOVA. **(D)** No significant differences were observed between diagnostic groups’ Aβ40 peripheral levels. **(E)** No significant differences were observed between diagnostic groups’ Aβ42 peripheral levels. **(F)** There was a main effect of diagnosis on Aβ42/Aβ40 ratio (*p* = 0.028). Statistical analyses; GLM for test of main and interaction effects, One Way ANOVA with LSD *post hoc* for multiple comparison, *p* ≤ 0.05 cut-off. ^∗^*p* ≤ 0.05, ^∗∗^*p* ≤ 0.01 vs. control. FABP3, Fatty acid binding protein 3; GFAP, Glial Fibrillary Acid Protein; Aβ, Amyloid-β.

We investigated several peripheral protein biomarkers in the same cohort to compare our lipidomic approach with immunoassay platforms. We investigated plasma levels of FABP3, GFAP, and Aβ fragments Aβ38, Aβ40, Aβ42, and Aβ42/Aβ40 ratio (Figures 4A–F). Preliminary analyses found a significant main effect of diagnosis for Aβ38 (*F*_(3)_ = 2.778, *p* = 0.045) and Aβ42/Aβ40 (*F*_(3)_ = 3.154, *p* = 0.028). *Post hoc* analysis did not find Aβ38 levels to be significantly different between groups. While the ratio of Aβ42 to Aβ40 was significantly different between the PTSD group vs. mTBI and mTBI + PTSD groups (*p* ≤ 0.05, Figure 4F).

## Discussion

Applications of lipidomics technology to profile plasma lipids of 120 active duty soldiers showed that several lipid classes differ by diagnostic classification in these military cohorts. We also report an influence of *APOE* ε4 carrier status on the association between lipid classes and mTBI and PTSD diagnoses. In particular, there was a significant interaction between both mTBI and mTBI + PTSD diagnostic groups and the ε4 carrier status on diverse lipid species, where ε4− participants with mTBI + PTSD showed the most pronounced changes in specific TG and DG species compared to controls. While the exact mechanisms behind peripheral lipid changes in response to CNS insults are yet to be fully elucidated, differences in these lipid signatures at non-acute timepoints, with inclusion of *APOE* genotyping, could help develop a lipid biomarker signature to assist clinicians with a differential diagnosis of these conditions.

Differential diagnosis of mTBI and PTSD is currently problematic due to symptom overlap and frequent comorbidity of these disorders, in both military cohorts and the general population (Laskowitz and Grant, 2016). Consistent with these findings, we observed shared symptomatology between TBI and PTSD using the NSI, where higher endorsement of symptoms generally thought to be associated with mTBI were reported among participants with PTSD in agreement with previous reports using the NSI (King et al., 2012; Porter et al., 2018). Although, NSI scores were somewhat lower in the mTBI group compared to the PTSD group, this might be because most individuals are asymptomatic within a few days following an mTBI (Kelly et al., 2012). Poorer sleep quality was also reported by participants with TBI and PTSD, which is a hallmark of both conditions (Gilbert et al., 2015). There were no significant differences between the controls and the diagnostic groups for stress and neurocognitive scores, which is not surprising given that the participants were young (mid-twenties) and more severe cases would not have been available due to injury or behavioral health issues identified at post-deployment medical screening. These observations further highlight the need for more objective biomarker measurements in TBI and PTSD.

Protein biomarkers currently used for TBI relate to either structural components of the brain (GFAP, neurofilaments, tau) or act as indicators of BBB injury (S100b, NSE) (Dadas et al., 2018). Therefore, these are likely to be more useful during the acute phases of the injury as a direct consequence of the physical damage but may not be as informative during sub-acute and chronic timepoints when biological changes likely reflect secondary consequences of injury, and the BBB is relatively intact, limiting their ability to enter the peripheral circulation. This is especially problematic since the initial features of injury do not necessarily correlate with long-term outcomes for patients (Dadas et al., 2018), particularly in mild injuries where immediate biological effects are subtle, often undetectable, and may only manifest as secondary consequences of the initial insult (Prince and Bruhns, 2017). Therefore, biomarkers associated with mTBI or PTSD could improve diagnosis to ensure appropriate care and management of these conditions. Although neuroimaging has been an invaluable tool for improving our understanding of TBI- and PTSD-related pathophysiology (Dadas et al., 2018; Dretsch et al., 2019) it is still limited in its utility over clinical measures (Dretsch et al., 2017a, b). Further, it is important to develop both cost effective and time efficient tools to track TBI-related changes while minimizing inconvenience to both patients and clinical staff. In our study, we did not observe any difference in protein markers, which appeared highly variable. This could be because some of these markers as previously discussed may be associated with more severe and acute injury, which were not present in our military sample, and therefore were not significantly elevated in our cohorts. Further, plasma levels of these protein biomarkers were within the range of control levels reported by others (Missler et al., 1998; Song et al., 2011; Catalucci et al., 2015).

Blood lipids are promising biomarkers as they are abundant, especially neutral lipids such as TG, DG, and CE which are present in the order of several μM to mM in blood (Burla et al., 2018), making them easily detectable. Lipid alterations have been reported for TBI and PTSD as well as other neuronal disorders, both centrally and peripherally (Adibhatla and Hatcher, 2007; Mellon et al., 2019), where hundreds of different molecular species are present and can inform on metabolic state (Burla et al., 2018). We examined total levels, the degree of unsaturation and chain lengths of lipid classes, as these can inform on lipid processes such as saturation and elongation, which may be disturbed following dysregulation of normal brain processes after TBI and PTSD. We also examined lipid species to determine whether specific lipid signatures could differentiate our three diagnostic groups. Functional magnetic resonance imaging and positron emission tomography have suggested metabolic changes in TBI (Dadas et al., 2018) and we have previously shown in these cohorts that several classes of plasma phospholipids including phosphatidylcholine (PC), lysoPC, phosphatidylethanolamine (PE), and lysoPE as well as sphingomyelin (SM) were all decreased in mTBI, PTSD, and mTBI + PTSD groups compared to controls (Emmerich et al., 2016). In the current study, we examined PS an additional PL as well as neutral lipids generally found in plasma lipid-rich lipoprotein particles responsible for transporting these lipids between various tissue including the brain and liver (Jonas and Phillips, 2008; Tracey et al., 2018). We also examined SM-related lipids, such as Cer and HexCer. While the exact mechanisms of changes in peripheral lipids after TBI and PTSD remain to be investigated, our present data show peripheral levels of TG, DG, CE, Cer, HexCer, and PS lipid classes to be differentially altered in mTBI, PTSD, and mTBI + PTSD groups which suggest a role of peripheral lipids, possibly requiring their transport into the brain for reparative and metabolic processes (Yu et al., 2011; Burla et al., 2018; Tracey et al., 2018). While in our previous study, PL differences were seen between controls and mTBI, PTSD, and mTBI + PTSD (Emmerich et al., 2016), in our current study, differences between controls and mTBI as well as mTBI + PTSD were notable for TG and DG and other individual species while only a few species differed between PTSD and controls and no class-wide differences were found in that group in agreement with what has been described by others (Jendrièko et al., 2009; Jergoviæ et al., 2015).

Different *APOE* isoforms differentially affect plasma lipids (Jonas and Phillips, 2008; Rasmussen, 2016). In particular, the presence of the ε4 allele is associated with impaired lipid transport in the periphery and the brain (Marais, 2019). Additionally, ε4 carriers have poorer outcomes after insults to the brain (Mota et al., 2017). In examining the influence of ε4 carrier status on the association between lipids and the diagnosis of TBI and PTSD, the greatest number of peripheral lipid changes were seen in the ε4− mTBI + PTSD group, while this was not the case in the ε4 + mTBI + PTSD group, indicating a differential response to injury in the presence of ε4 allele. Changes in TG and DG levels could reflect increased requirement for lipids to fuel the repair processes within the brain after injury (Tracey et al., 2018). Furthermore, TG and DG that contain certain long-chain PUFA require transport into the brain since approximately 35% of PUFA cannot be synthesized *de novo* and are acquired through diet (Liu et al., 2015). Differential profiles of PUFA with TG and DG following injury by ε4 carrier status may suggest differential uptake of these lipids by the brain. Further, as Cer are central to the sphingolipid metabolism (Burla et al., 2018), and Hexcer are precursors to gangliosides which are abundant in the brain (Yu et al., 2011), blood changes in these lipids may be indicative of altered sphingolipid metabolism after injury. There was also an increase in several PS lipid species in all ε4− diagnostic groups. Phosphatidylserine is enriched in inner cell membranes and is involved in numerous important signaling pathways, apoptotic cell clearance, coagulation and response to histamine secretion (Leventis and Grinstein, 2010; Kim et al., 2014; Brelstaff et al., 2018). Hence, examination of the influence of ε4 on these lipids in relation to injury warrants further investigation.

While these lipid profile suggest their differential association with diagnosis and ε4 status, small sample size remains a limitation. Although precautions were taken to minimize sampling bias (i.e., evaluation of confounders and outliers), these studies require further validation in larger cohorts of other military populations. Further blood lipids can be affected by diet, lifestyle choices and chronic health conditions, such as cardiovascular risk factors (i.e., hypertension, high cholesterol, and diabetes). However, medication use data in our cohorts suggest that this was a healthy young cohort as the prevalence of cardiovascular medication was relatively low and our analyses showed that these were randomly distributed between diagnostic groups. Antidepressants was the only therapeutic category found to be unequally distributed across groups, but further analyses showed this did not confound the relationship between lipids and diagnosis in this study. Although limited data are available on dietary variations and lipid profiles, a recent study by Begum et al. (2016) examining inter- and intra-person variation in blood lipids showed that daily diet and time of the day only affected 8 out of 196 lipids which were examined. Specifically, they showed that PS, Cer, and HexCer lipids were not significant sources of variations over the course of the day (Begum et al., 2016). Unfortunately, data on diet and other lifestyle factors were unavailable in this cohort but given that they were from a military cohort, we may expect them to have similar lifestyles and diet at the time of deployment, as was suggested by our basic demographic data. Further, our semi-quantitative lipid data in control groups is consistent with what has been previously reported (Quehenberger et al., 2010). Therefore, our controls appear to be representative of the general population and should be able to control for some of the inter- and intra-individual variation expected. Additionally, our neurobehavioral and health questionnaire data suggests that these cohort are representative of military TBI and PTSD cohorts that have been previously described (King et al., 2012; Gilbert et al., 2015; Porter et al., 2018).

Expanding on our previous work these findings suggest that a panel of peripheral lipids may be useful to help differentiate mTBI from PTSD at subacute to chronic timepoints post-insult. Further, these data highlight the importance of knowing *APOE* genotype when examining and interpreting patients’ blood lipid levels as we show that *APOE* genotype, specifically carrier status for the ε4 allele, has an important (sometimes opposing) influence when stratifying blood lipid profiles by diagnosis, which is consistent with our work in these cohorts examining different PL. Traumatic brain injury sequelae and PTSD are heterogeneous and currently assessed using criteria that are not necessarily well correlated with pathology, which is why using a lipidomics approach to examine a panel of lipid biomarkers could help pave the way toward building a better biological phenotype of these disorders. If changes seen in these peripheral lipids are replicated in other cohorts these could become useful clinical biomarkers in addition to available diagnostic tools. Longitudinal studies will be necessary to elucidate the potential of peripheral lipids to predict outcome and recovery after injury. Lastly, changes in composite variables and individual species could be further characterized in animal models, tissues and cells to identify altered networks which could, in turn, identify key regulators to target with potential therapeutics as we and others keep characterizing lipid profiles in neurological disease.

## Data Availability Statement

The raw data supporting the conclusions of this article will be made available by the authors, without undue reservation, to any qualified researcher.

## Ethics Statement

The study was approved by the Institutional Review Board at Headquarters U.S. Army Medical Research and Materiel Command, Fort Detrick, MD (HQ United States MRMC IRB), and the protocol and procedures were carried out in accordance with the Declaration of Helsinki. Soldiers participated on a voluntary basis after receiving a study brief. An IRB approved informed consent was provided in accordance with international conference on harmonization guidelines and a signed consent was obtained from all participants in the presence of an ombudsperson. Approval was also obtained from brigade commanders.

## Author Contributions

CH wrote the manuscript, performed the immunoassay experiments, and analyzed the data. SO, HL, TD, and AN assisted with the experiments. AC and JE set-up the lipidomic assay. MD and FC conceived the original study design for biomarker investigation in these populations. MD, FC, and SF were involved in obtention of samples and processing. MM and FC provided institutional support. LA designed the experiments. LA, JE, MD, and FC reviewed and edited the manuscript.

## Disclaimer

Material has been reviewed by the Walter Reed Army Institute of Research. There is no objection to its presentation and/or publication. The opinions or assertions contained herein are the private views of the author, and are not to be construed as official, or as reflecting true views of the Department of the Army or the Department of Defense. The investigators have adhered to the policies for protection of human subjects as prescribed in AR 70–25.

## Conflict of Interest

The authors declare that the research was conducted in the absence of any commercial or financial relationships that could be construed as a potential conflict of interest.
