# Supplementary material for: Plasma Lipidomic Analyses in Cohorts With mTBI and/or PTSD Reveal Lipids Differentially Associated With Diagnosis and APOE ε4 Carrier Status
Source: Front Physiol. 2020 Jan 31;11:12. doi: 10.3389/fphys.2020.00012 (PMC7005602; doi:10.3389/fphys.2020.00012)
Supplement: Supplementary file 1 [file Table_1.docx]

Supplementary Material

Table 1. Basic demographics of the study population. Statistical analyses; GLM for test of main and interaction effects, One Way ANOVA with LSD *post-hoc* for multiple comparison. Bolded numbers indicate significant between group differences against their respective control. For medication use 5 subjects on multiple medications (*n*=1 in ε4-control, *n*=1 in ε4+control, *n*=1 in ε4-mTBI, *n*=2 in ε4-PTSD). NCO: Non-commissioned officer.

|  |  | Control | | mTBI | | PTSD | | mTBI+PTSD | |
| --- | --- | --- | --- | --- | --- | --- | --- | --- | --- |
| APOE ε4 |  | - | + | - | + | - | + | - | + |
| Numbers |  | *n*=37 | *n*=15 | *n*=16 | *n*=5 | *n*=24 | *n*=10 | *n*=8 | *n*=5 |
| Age (average ± SD) |  | 27±7 | 27±8 | 25±8 | 30±11 | 28±4 | 23±4 | 30±7 | 29±4 |
| Education (average years ± SD) |  | 13±1 | 13±1 | 13±1 | 13±2 | 13±1 | 13±1 | **14±2** | **14±1** |
| Mother’s education (average years ± SD) |  | 13±3 | 14±2 | 14±3 | 13±2 | 13±2 | 14±2 | 14±3 | 13±3 |
| Race (*n*) | Black | 1 | 1 | 1 | 1 | 2 | 2 | 0 | 0 |
|  | White | 29 | 10 | 11 | 4 | 18 | 7 | 7 | 3 |
|  | Pacific Islander | 2 | 0 | 1 | 0 | 0 | 1 | 1 | 0 |
|  | Hispanic/Latino | 4 | 1 | 2 | 0 | 3 | 0 | 0 | 2 |
|  | Native American | 1 | 0 | 0 | 0 | 0 | 0 | 0 | 0 |
|  | Asian | 0 | 1 | 0 | 0 | 1 | 0 | 0 | 0 |
|  | Other | 0 | 2 | 1 | 0 | 0 | 0 | 0 | 0 |
| Total deployments (*n*) | 0 | 11 | 6 | 5 | 2 | 6 | 5 | 0 | 0 |
|  | 1 | 15 | 6 | 5 | 0 | 12 | 3 | 2 | 1 |
|  | 2 | 7 | 2 | 6 | 3 | 2 | 2 | 6 | 3 |
|  | 3 | 4 | 1 | 0 | 0 | 2 | 0 | 0 | 0 |
|  | 4 | 0 | 0 | 0 | 0 | 2 | 0 | 0 | 1 |
| Paygrade | Junior enlisted | 7 | 4 | 4 | 2 | 8 | 4 | 0 | 0 |
|  | NCO | 30 | 10 | 12 | 2 | 14 | 6 | 8 | 5 |
|  | Senior NCO | 0 | 1 | 0 | 1 | 2 | 0 | 0 | 0 |
| Medication use (*n*) | None | 33 | 14 | 15 | 5 | 14 | 10 | 5 | 4 |
|  | Anti-depressants | 0 | 0 | 1 | 0 | **5** | 0 | 1 | 0 |
|  | Anti-inflammatories | 1 | 1 | 0 | 0 | 1 | 0 | 1 | 1 |
|  | Analgesics | 0 | 0 | 0 | 0 | 1 | 0 | 1 | 0 |
|  | Sedatives & hypnotics | 0 | 0 | 1 | 0 | 0 | 0 | 1 | 0 |
|  | Anti-bacterial | 1 | 0 | 0 | 0 | 1 | 0 | 0 | 0 |
|  | Cardiovascular medications | 2 | 0 | 0 | 0 | 3 | 0 | 0 | 0 |
|  | Gastrointestinal agents | 1 | 0 | 0 | 0 | 2 | 0 | 0 | 0 |
|  | Allergy medication | 0 | 1 | 0 | 0 | 0 | 0 | 0 | 0 |

Table 2. Psychological health questionnaires, neurobehavioral symptoms and neurocognitive data of the study population. Statistical analyses; GLM for test of main and interaction effects, One Way ANOVA with LSD *post-hoc* for multiple comparison. Tests that yielded significant between group differences are presented first. Bolded numbers indicate a significant difference compared to control. NSI: Neurobehavioral Symptoms Inventory, ESS: Epworth Sleep Scale, PSQI: Pittsburg Sleep Quality Index, ZDS: Zung Depression Scale, AUDIT: Alcohol Use Dependency Identification Test, SAS: Zung Self-Rating Anxiety Scale, PSS: Perceived Stress Scale, CNS-VS: Central Nervous System – Vital Signs.

|  |  | Control | mTBI | PTSD | mTBI+PTSD |
| --- | --- | --- | --- | --- | --- |
| Numbers |  | *n*=52 | *n*=21 | *n*=34 | *n*=13 |
| NSI (average ± SD) |  | 6±7 | **13±12** | **28±15** | **26±17** |
| PSQI (*n*) | Normal (≤3) | 12 | **5** | **3** | **2** |
|  | Poor (> 4) | 40 | **16** | **31** | **11** |
| ESS (*n*) | Normal (0-10) | 39 | 15 | **17** | 12 |
|  | Mild (11-12) | 5 | 1 | **1** | 0 |
|  | Moderate (13-15) | 5 | 2 | **10** | 0 |
|  | Severe (16-24) | 3 | 3 | **6** | 1 |
| ZDS (*n*) | Normal (≤49) | 52 | 20 | **22** | 13 |
|  | Mild (50-59) | 0 | 1 | **9** | 0 |
|  | Moderate (60-69) | 0 | 0 | **3** | 0 |
|  | Severe (≥70) | 0 | 0 | **0** | 0 |
| SAS (*n*) | Normal (20-44) | 52 | **19** | **26** | **11** |
|  | Mild to moderate (45-59) | 0 | **2** | **8** | **2** |
|  | Moderate to severe (60-74) | 0 | **0** | **0** | **0** |
|  | Extreme (≥75) | 0 | **0** | **0** | **0** |
| AUDIT (*n*) | Normal (≤7) | 33 | 12 | 16 | 5 |
|  | Harmful/hazardous (≥8) | 17 | 7 | 14 | 7 |
|  | Dependence (≥15) | 2 | 2 | 4 | 1 |
| PSS (*n*) | Low (0-13) | 8 | 3 | 0 | 1 |
|  | Moderate (14-26) | 19 | 7 | 16 | 3 |
|  | High (27-40) | 25 | 11 | 18 | 9 |
| CNS-VS (average ± SD) | Verbal memory | 94±22 | 98±19 | 90±20 | 88±24 |
|  | Information processing speed | 96±22 | 90±13 | 90±13 | 95±18 |
|  | Complex attention | 85±24 | 81±25 | 81±24 | 79±26 |
|  | Cognitive flexibility | 94±22 | 90±23 | 90±20 | 94±24 |
|  | Reaction time | 99±24 | 94±18 | 93±13 | 99±21 |
|  | Executive function | 97±22 | 92±23 | 92±20 | 97±24 |

**Figure S1**. Peripheral PS levels in mTBI, PTSD and mTBI+PTSD diagnostic groups with differential *APOE* genotype. **(A)** No significant differences were found in PS unsaturation states between groups. **(B)** No significant differences were found in PS chain length states between groups. Statistical analyses; MLM with LSD *post-hoc*, p≤0.05 cut-off. PS: Phosphatidylserines, SFA: saturated fatty acids, MUFA: Mono-unsaturated fatty acids, PUFA: poly-unsaturated fatty acids.

Table 3. Average ± SEM TG levels (µM) of sum composition species examined in all diagnostic groups with ε4 stratification. This data was QC normalized and semi-quantified using a 15:0-18:1(d7)-15:0 TG IS. The average number per group is shown for each diagnostic group and ε4 subgroups.

|  | **Triglycerides (µM)** | | | | | | | | | | | | | | | |
| --- | --- | --- | --- | --- | --- | --- | --- | --- | --- | --- | --- | --- | --- | --- | --- | --- |
|  | **Control** | | | | **mTBI** | | | | **PTSD** | | | | **mTBI+PTSD** | | | |
|  | n= 52 | | | | n= 21 | | | | n= 34 | | | | n= 13 | | | |
| **APOE ε4** | **-** | | **+** | | **-** | | **+** | | **-** | | **+** | | **-** | | **+** | |
|  | n= 37 | | n= 15 | | n= 16 | | n= 5 | | n= 24 | | n= 10 | | n= 8 | | n= 5 | |
|  | **Mean** | **SEM** | **Mean** | **SEM** | **Mean** | **SEM** | **Mean** | **SEM** | **Mean** | **SEM** | **Mean** | **SEM** | **Mean** | **SEM** | **Mean** | **SEM** |
| **TG(46:0)** | 8.46 | 1.04 | 6.26 | 0.53 | 8.78 | 0.76 | 4.39 | 0.56 | 7.02 | 0.66 | 7.66 | 0.68 | 10.22 | 1.59 | 6.79 | 1.22 |
| **TG(46:1)** | 20.16 | 2.05 | 14.51 | 1.58 | 22.58 | 2.73 | 10.79 | 1.16 | 13.96 | 1.40 | 20.04 | 2.45 | 30.84 | 7.90 | 16.90 | 4.35 |
| **TG(48:0)** | 9.33 | 1.26 | 6.99 | 0.51 | 8.01 | 0.66 | 5.03 | 0.70 | 7.95 | 0.56 | 7.27 | 0.39 | 9.15 | 1.19 | 8.78 | 1.93 |
| **TG(48:1)** | 30.81 | 2.85 | 21.23 | 1.80 | 26.96 | 2.52 | 15.25 | 1.30 | 25.06 | 2.59 | 27.26 | 2.64 | 47.22 | 11.60 | 25.82 | 5.25 |
| **TG(48:2)** | 47.35 | 3.48 | 36.28 | 3.19 | 48.09 | 5.14 | 31.29 | 2.72 | 39.31 | 3.98 | 44.79 | 4.43 | 66.06 | 17.84 | 39.45 | 8.38 |
| **TG(50:0)** | 8.33 | 0.98 | 6.85 | 0.54 | 6.78 | 0.53 | 5.32 | 0.62 | 7.48 | 0.50 | 6.59 | 0.44 | 7.75 | 1.02 | 9.71 | 2.67 |
| **TG(50:1)** | 37.67 | 2.89 | 28.74 | 2.40 | 28.47 | 2.35 | 21.23 | 2.05 | 34.13 | 2.52 | 32.28 | 3.27 | 62.12 | 12.28 | 37.41 | 7.33 |
| **TG(50:2)** | 89.32 | 5.67 | 72.64 | 4.92 | 73.94 | 5.07 | 59.05 | 3.07 | 82.24 | 6.57 | 75.65 | 5.53 | 133.67 | 29.16 | 83.03 | 13.64 |
| **TG(50:3)** | 92.89 | 5.25 | 80.89 | 6.19 | 84.93 | 6.52 | 79.34 | 7.04 | 84.77 | 6.87 | 81.97 | 6.65 | 120.67 | 27.33 | 86.86 | 15.02 |
| **TG(50:4)** | 47.48 | 3.15 | 43.45 | 3.83 | 46.91 | 4.35 | 53.04 | 7.03 | 41.34 | 4.03 | 43.56 | 3.25 | 61.48 | 14.51 | 45.24 | 10.10 |
| **TG(50:5)** | 12.45 | 0.94 | 10.87 | 1.05 | 12.71 | 1.35 | 16.03 | 3.25 | 10.40 | 1.13 | 11.54 | 0.93 | 14.68 | 3.70 | 11.16 | 2.74 |
| **TG(50:6)** | 2.36 | 0.25 | 1.96 | 0.27 | 2.72 | 0.40 | 3.29 | 0.72 | 1.80 | 0.26 | 2.09 | 0.24 | 2.80 | 0.87 | 1.84 | 0.47 |
| **TG(52:0)** | 10.39 | 1.31 | 7.71 | 0.68 | 9.39 | 1.17 | 10.07 | 1.91 | 10.77 | 0.87 | 7.33 | 0.39 | 12.53 | 2.73 | 9.49 | 1.73 |
| **TG(52:1)** | 9.88 | 1.05 | 10.13 | 1.65 | 7.12 | 1.09 | 5.51 | 1.01 | 8.73 | 0.75 | 9.83 | 1.49 | 15.56 | 2.13 | 13.80 | 4.21 |
| **TG(52:2)** | 100.08 | 6.26 | 94.01 | 9.42 | 78.30 | 5.63 | 79.30 | 8.29 | 96.37 | 6.47 | 95.11 | 11.22 | 159.32 | 26.40 | 104.71 | 19.34 |
| **TG(52:3)** | 224.72 | 12.54 | 212.17 | 14.01 | 196.70 | 11.05 | 197.91 | 11.61 | 215.09 | 13.92 | 200.34 | 13.09 | 315.10 | 52.83 | 236.83 | 39.01 |
| **TG(52:4)** | 229.05 | 14.90 | 222.39 | 17.63 | 208.41 | 13.03 | 222.86 | 15.02 | 209.04 | 14.57 | 198.74 | 12.73 | 288.48 | 49.97 | 220.20 | 36.29 |
| **TG(52:5)** | 86.38 | 6.36 | 85.61 | 7.43 | 80.06 | 5.69 | 98.20 | 9.83 | 76.32 | 6.08 | 77.74 | 5.23 | 108.81 | 21.19 | 85.05 | 17.05 |
| **TG(52:6)** | 19.04 | 1.57 | 17.65 | 1.61 | 18.20 | 1.67 | 25.10 | 4.22 | 16.11 | 1.53 | 17.66 | 1.35 | 21.60 | 4.57 | 18.10 | 4.20 |
| **TG(52:7)** | 1.06 | 0.19 | 0.85 | 0.22 | 1.70 | 0.34 | 2.38 | 0.95 | 1.01 | 0.21 | 1.06 | 0.19 | 2.02 | 0.65 | 1.46 | 0.45 |
| **TG(53:3)** | 5.41 | 0.38 | 5.06 | 0.50 | 4.52 | 0.34 | 4.81 | 0.50 | 4.71 | 0.33 | 5.27 | 0.55 | 8.10 | 1.11 | 4.46 | 0.81 |
| **TG(53:4)** | 5.85 | 0.44 | 5.61 | 0.50 | 5.10 | 0.37 | 5.59 | 0.34 | 4.91 | 0.34 | 5.41 | 0.38 | 7.60 | 1.15 | 4.83 | 0.83 |
| **TG(54:0)** | 7.64 | 1.14 | 4.79 | 0.50 | 7.92 | 1.71 | 9.75 | 2.78 | 9.69 | 1.59 | 4.57 | 0.22 | 14.01 | 5.11 | 5.08 | 0.55 |
| **TG(54:1)** | 1.43 | 0.22 | 1.88 | 0.45 | 1.21 | 0.31 | 0.91 | 0.22 | 1.14 | 0.11 | 1.35 | 0.21 | 1.59 | 0.17 | 2.30 | 1.02 |
| **TG(54:2)** | 8.82 | 0.92 | 12.54 | 3.25 | 6.71 | 1.26 | 6.09 | 1.20 | 7.73 | 0.71 | 11.23 | 2.59 | 14.25 | 1.85 | 10.92 | 3.06 |
| **TG(54:3)** | 42.08 | 3.98 | 46.85 | 8.12 | 30.60 | 3.45 | 34.23 | 4.24 | 36.45 | 2.78 | 43.21 | 6.88 | 56.67 | 7.36 | 44.25 | 9.69 |
| **TG(54:4)** | 87.06 | 7.57 | 85.93 | 9.90 | 67.66 | 5.47 | 77.78 | 5.68 | 71.36 | 4.91 | 78.68 | 7.22 | 121.02 | 19.73 | 84.68 | 16.71 |
| **TG(54:6)** | 96.97 | 11.93 | 90.50 | 9.74 | 84.05 | 6.04 | 104.91 | 12.22 | 69.00 | 4.93 | 86.31 | 8.11 | 133.13 | 27.46 | 77.27 | 15.07 |
| **TG(54:7)** | 36.87 | 5.75 | 34.42 | 3.56 | 33.21 | 2.40 | 46.92 | 7.96 | 23.93 | 2.11 | 33.82 | 3.40 | 54.02 | 13.91 | 31.17 | 7.30 |
| **TG(55:6)** | 0.77 | 0.04 | 0.72 | 0.04 | 0.87 | 0.08 | 0.88 | 0.09 | 0.72 | 0.04 | 0.81 | 0.06 | 1.14 | 0.16 | 0.95 | 0.16 |
| **TG(56:1)** | 0.18 | 0.03 | 0.19 | 0.04 | 0.29 | 0.12 | 0.15 | 0.03 | 0.17 | 0.02 | 0.22 | 0.06 | 0.24 | 0.04 | 0.14 | 0.05 |
| **TG(56:2)** | 0.48 | 0.07 | 0.54 | 0.13 | 0.58 | 0.21 | 0.28 | 0.05 | 0.37 | 0.04 | 0.80 | 0.25 | 0.68 | 0.10 | 0.47 | 0.15 |
| **TG(56:3)** | 1.02 | 0.14 | 1.00 | 0.20 | 0.95 | 0.22 | 0.70 | 0.11 | 0.86 | 0.08 | 1.54 | 0.46 | 1.85 | 0.33 | 1.01 | 0.25 |
| **TG(56:4)** | 2.23 | 0.26 | 1.82 | 0.23 | 1.71 | 0.24 | 1.71 | 0.21 | 1.79 | 0.15 | 2.13 | 0.36 | 3.45 | 0.54 | 2.09 | 0.48 |
| **TG(56:5)** | 8.00 | 0.62 | 7.35 | 0.71 | 6.87 | 0.50 | 8.11 | 0.65 | 7.31 | 0.53 | 7.76 | 0.94 | 13.26 | 2.15 | 8.36 | 1.32 |
| **TG(56:6)** | 19.04 | 1.11 | 18.86 | 1.17 | 18.54 | 0.92 | 21.41 | 1.53 | 18.58 | 1.21 | 18.26 | 1.35 | 26.71 | 3.64 | 24.62 | 3.17 |
| **TG(56:7)** | 25.39 | 1.37 | 24.31 | 1.46 | 27.79 | 1.83 | 29.65 | 2.85 | 25.43 | 1.57 | 23.70 | 1.91 | 37.22 | 5.18 | 32.24 | 3.53 |
| **TG(58:2)** | 0.20 | 0.04 | 0.20 | 0.05 | 0.43 | 0.21 | 0.11 | 0.02 | 0.14 | 0.02 | 0.49 | 0.18 | 0.28 | 0.05 | 0.13 | 0.04 |
| **TG(58:6)** | 1.04 | 0.10 | 1.04 | 0.18 | 0.89 | 0.11 | 1.17 | 0.15 | 0.99 | 0.10 | 1.16 | 0.24 | 2.13 | 0.39 | 1.13 | 0.19 |
| **TG(58:7)** | 2.52 | 0.17 | 2.39 | 0.21 | 2.50 | 0.18 | 2.88 | 0.27 | 2.36 | 0.16 | 2.32 | 0.18 | 4.23 | 0.63 | 3.34 | 0.41 |
| **TG(58:8)** | 4.50 | 0.28 | 4.10 | 0.24 | 4.97 | 0.49 | 5.29 | 0.51 | 4.30 | 0.26 | 4.28 | 0.36 | 7.33 | 1.29 | 5.22 | 0.47 |
| **TG(58:9)** | 5.22 | 0.30 | 4.85 | 0.27 | 6.08 | 0.48 | 6.36 | 0.59 | 5.41 | 0.30 | 5.28 | 0.49 | 7.78 | 1.03 | 6.06 | 0.55 |

Table 4. Average ± SEM DG levels (µM) of sum composition species examined in all diagnostic groups with ε4 stratification. This data was QC normalized and semi-quantified using a 15:0-18:1(d7) DG IS. The average number per group is shown for each diagnostic group and ε4 subgroups.

|  | **Diglycerides (µM)** | | | | | | | | | | | | | | | | |
| --- | --- | --- | --- | --- | --- | --- | --- | --- | --- | --- | --- | --- | --- | --- | --- | --- | --- |
|  | **Control** | | | | **mTBI** | | | | **PTSD** | | | | **mTBI+PTSD** | | | | |
|  | n= 49 | | | | n= 21 | | | | n= 34 | | | | n= 13 | | | | |
| **APOE ε4** | **-** | | **+** | | **-** | | **+** | | **-** | | **+** | | **-** | | **+** | | |
|  | n= 34 | | n= 15 | | n= 16 | | n= 5 | | n= 24 | | n= 10 | | n= 8 | | n= 5 | | |
|  | **Mean** | **SEM** | **Mean** | **SEM** | **Mean** | **SEM** | **Mean** | **SEM** | **Mean** | **SEM** | **Mean** | **SEM** | **Mean** | **SEM** | **Mean** | **SEM** |  |
| **DG(30:0)** | 23.52 | 1.21 | 20.79 | 1.06 | 20.77 | 1.26 | 32.55 | 3.37 | 24.12 | 1.22 | 22.22 | 1.35 | 28.03 | 2.70 | 21.23 | 2.18 |  |
| **DG(32:0)** | 143.09 | 5.27 | 140.16 | 6.87 | 127.41 | 9.43 | 186.10 | 12.94 | 164.60 | 8.48 | 136.40 | 5.72 | 177.27 | 19.51 | 157.58 | 12.16 |  |
| **DG(32:1)** | 1.71 | 0.08 | 1.85 | 0.20 | 2.03 | 0.28 | 1.56 | 0.12 | 1.66 | 0.11 | 1.81 | 0.10 | 2.27 | 0.52 | 1.80 | 0.28 |  |
| **DG(32:3)** | 0.18 | 0.01 | 0.13 | 0.01 | 0.17 | 0.02 | 0.22 | 0.02 | 0.16 | 0.01 | 0.13 | 0.01 | 0.19 | 0.03 | 0.16 | 0.04 |  |
| **DG(34:0)** | 237.70 | 20.86 | 186.05 | 9.86 | 237.27 | 40.36 | 381.83 | 66.35 | 330.25 | 42.64 | 205.17 | 15.51 | 445.15 | 81.65 | 233.99 | 21.78 |  |
| **DG(34:1)** | 8.62 | 0.64 | 9.00 | 0.58 | 8.57 | 1.08 | 9.33 | 1.92 | 10.53 | 0.87 | 8.37 | 0.43 | 13.87 | 2.43 | 10.67 | 1.43 |  |
| **DG(34:2)** | 5.55 | 0.30 | 5.66 | 0.30 | 5.18 | 0.35 | 5.11 | 0.39 | 5.71 | 0.38 | 5.06 | 0.28 | 7.62 | 1.65 | 6.56 | 0.91 |  |
| **DG(34:3)** | 0.99 | 0.06 | 1.02 | 0.06 | 1.07 | 0.09 | 1.10 | 0.12 | 1.03 | 0.09 | 0.93 | 0.06 | 1.44 | 0.31 | 1.20 | 0.17 |  |
| **DG(36:2)** | 12.51 | 0.79 | 14.51 | 1.13 | 11.34 | 1.08 | 12.81 | 2.03 | 13.64 | 0.90 | 11.62 | 0.78 | 16.30 | 2.85 | 15.64 | 2.72 |  |
| **DG(36:3)** | 10.89 | 0.72 | 12.22 | 0.86 | 9.56 | 0.62 | 11.52 | 0.83 | 11.08 | 0.80 | 9.78 | 0.74 | 13.49 | 2.72 | 13.69 | 2.14 |  |
| **DG(36:4)** | 4.13 | 0.40 | 4.51 | 0.33 | 4.11 | 0.31 | 5.12 | 0.55 | 3.98 | 0.29 | 3.45 | 0.31 | 5.52 | 0.95 | 5.34 | 0.89 |  |
| **DG(36:5)** | 0.39 | 0.03 | 0.39 | 0.04 | 0.40 | 0.05 | 0.52 | 0.05 | 0.42 | 0.04 | 0.34 | 0.04 | 0.42 | 0.08 | 0.39 | 0.06 |  |
| **DG(38:0)** | 5.30 | 0.45 | 4.44 | 0.30 | 5.82 | 0.89 | 7.50 | 1.13 | 6.68 | 0.71 | 3.80 | 0.25 | 9.62 | 1.40 | 4.14 | 0.52 |  |
| **DG(38:3)** | 0.39 | 0.03 | 0.42 | 0.04 | 0.32 | 0.04 | 0.33 | 0.04 | 0.35 | 0.03 | 0.30 | 0.02 | 0.37 | 0.12 | 0.42 | 0.09 |  |
| **DG(38:4)** | 0.46 | 0.03 | 0.53 | 0.04 | 0.43 | 0.05 | 0.40 | 0.06 | 0.51 | 0.05 | 0.39 | 0.04 | 0.46 | 0.14 | 0.62 | 0.12 |  |
| **DG(38:4)2*** | 0.26 | 0.01 | 0.30 | 0.02 | 0.26 | 0.02 | 0.21 | 0.02 | 0.32 | 0.03 | 0.19 | 0.01 | 0.23 | 0.04 | 0.28 | 0.05 |  |
| **DG(38:5)** | 0.89 | 0.05 | 0.97 | 0.07 | 0.85 | 0.07 | 0.81 | 0.05 | 1.02 | 0.07 | 0.93 | 0.11 | 0.99 | 0.22 | 1.45 | 0.28 |  |
| **DG(40:7)** | 0.78 | 0.07 | 0.52 | 0.06 | 0.75 | 0.11 | 0.76 | 0.11 | 0.87 | 0.10 | 0.57 | 0.04 | 0.54 | 0.10 | 1.64 | 0.51 |  |

* DG(34:2)2 indicates a second separate peak was found and integrated for that sum composition lipid.

Table 5. Average ± SEM CE and unesterified cholesterol levels (µM) of individual species examined in all diagnostic groups with ε4 stratification. This data was QC normalized and semi-quantified using 18:1(d7) Chol Ester IS for CE and Cholesterol (d7) IS for free cholesterol. The average number per group is shown for each diagnostic group and ε4 subgroups.

|  | **Cholesterol and cholesterol esters (µM)** | | | | | | | | | | | | | | | | |
| --- | --- | --- | --- | --- | --- | --- | --- | --- | --- | --- | --- | --- | --- | --- | --- | --- | --- |
|  | **Control** | | | | **mTBI** | | | | **PTSD** | | | | **mTBI+PTSD** | | | | |
|  | n= 50 | | | | n= 20 | | | | n= 33 | | | | n= 13 | | | | |
| **APOE ε4** | **-** | | **+** | | **-** | | **+** | | **-** | | **+** | | **-** | | **+** | | |
|  | n= 35 | | n= 15 | | n= 15 | | n= 5 | | n= 23 | | n= 10 | | n= 8 | | n= 5 | | |
|  | **Mean** | **SEM** | **Mean** | **SEM** | **Mean** | **SEM** | **Mean** | **SEM** | **Mean** | **SEM** | **Mean** | **SEM** | **Mean** | **SEM** | **Mean** | **SEM** |  |
| **Free Cholesterol** | 1655.15 | 67.70 | 1522.67 | 84.43 | 1631.11 | 85.21 | 1686.53 | 136.59 | 1801.63 | 77.74 | 1448.63 | 63.69 | 2080.28 | 161.62 | 1732.85 | 243.20 |  |
| **CE(14:1)** | 8.70 | 0.66 | 8.48 | 0.66 | 8.22 | 1.51 | 5.82 | 1.21 | 8.91 | 0.83 | 9.62 | 1.38 | 5.65 | 1.11 | 6.63 | 1.12 |  |
| **CE(15:0)** | 15.19 | 0.80 | 14.69 | 0.78 | 16.85 | 1.17 | 13.85 | 1.78 | 15.01 | 0.95 | 15.92 | 1.16 | 16.61 | 1.60 | 12.92 | 1.74 |  |
| **CE(16:1)** | 261.03 | 11.60 | 221.77 | 10.96 | 233.55 | 15.15 | 196.05 | 13.11 | 262.20 | 12.28 | 243.84 | 21.34 | 268.15 | 41.41 | 265.04 | 38.95 |  |
| **CE(17:1)** | 22.97 | 0.84 | 22.86 | 1.28 | 20.98 | 1.34 | 20.97 | 1.68 | 24.00 | 1.04 | 23.55 | 1.42 | 25.98 | 2.42 | 24.79 | 3.18 |  |
| **CE(18:1)** | 861.15 | 30.43 | 882.38 | 77.99 | 893.36 | 60.51 | 857.97 | 42.67 | 1008.16 | 50.94 | 827.85 | 49.07 | 1007.02 | 78.30 | 927.13 | 106.92 |  |
| **CE(18:2)** | 3265.26 | 88.02 | 2953.46 | 109.90 | 3209.01 | 157.56 | 3125.38 | 160.62 | 3524.06 | 140.73 | 3197.85 | 269.18 | 3378.51 | 268.07 | 3146.87 | 325.38 |  |
| **CE(18:3)** | 420.32 | 19.06 | 405.47 | 27.94 | 425.96 | 31.92 | 387.28 | 35.57 | 459.17 | 23.80 | 457.17 | 43.32 | 375.08 | 55.16 | 420.02 | 52.17 |  |
| **CE(20:1)** | 2.39 | 0.63 | 1.81 | 0.22 | 5.83 | 2.14 | 1.88 | 0.36 | 3.22 | 0.83 | 2.01 | 0.31 | 3.44 | 0.45 | 5.44 | 1.65 |  |
| **CE(20:3)** | 107.00 | 4.22 | 102.53 | 5.53 | 101.21 | 4.82 | 92.94 | 5.79 | 117.52 | 4.34 | 114.27 | 10.35 | 123.10 | 15.34 | 113.36 | 14.73 |  |
| **CE(20:4)** | 1333.83 | 44.88 | 1201.16 | 81.33 | 1446.87 | 87.32 | 1298.48 | 82.64 | 1530.35 | 69.07 | 1156.47 | 61.58 | 1338.55 | 134.57 | 1426.67 | 94.01 |  |
| **CE(20:5)** | 164.30 | 8.89 | 138.90 | 11.87 | 180.19 | 14.41 | 124.82 | 14.78 | 172.14 | 13.92 | 153.87 | 19.10 | 117.47 | 12.46 | 188.60 | 26.31 |  |
| **CE(22:5)** | 10.79 | 0.70 | 10.67 | 2.32 | 11.05 | 0.54 | 15.41 | 4.48 | 13.19 | 1.67 | 9.73 | 0.58 | 10.81 | 0.97 | 12.77 | 0.92 |  |
| **CE(22:6)** | 104.12 | 5.01 | 103.51 | 8.65 | 122.96 | 8.62 | 100.46 | 9.46 | 128.28 | 7.86 | 88.79 | 6.50 | 138.15 | 15.79 | 125.58 | 10.36 |  |

Table 6. Average ± SEM PS levels (µM) of sum composition species examined in all diagnostic groups with ε4 stratification. This data was QC normalized and semi-quantified using a 15:0-18:1(d7) PS IS. The average number per group is shown for each diagnostic group and ε4 subgroups.

|  | **Phosphatidylserines (µM)** | | | | | | | | | | | | | | | | |
| --- | --- | --- | --- | --- | --- | --- | --- | --- | --- | --- | --- | --- | --- | --- | --- | --- | --- |
|  | **Control** | | | | **mTBI** | | | | **PTSD** | | | | **mTBI+PTSD** | | | | |
|  | n= 49 | | | | n= 21 | | | | n= 33 | | | | n= 13 | | | | |
| **APOE ε4** | **-** | | **+** | | **-** | | **+** | | **-** | | **+** | | **-** | | **+** | | |
|  | n= 34 | | n= 15 | | n= 16 | | n= 5 | | n= 23 | | n= 10 | | n= 8 | | n= 5 | | |
|  | **Mean** | **SEM** | **Mean** | **SEM** | **Mean** | **SEM** | **Mean** | **SEM** | **Mean** | **SEM** | **Mean** | **SEM** | **Mean** | **SEM** | **Mean** | **SEM** |  |
| **PS(34:0)** | 2.17 | 0.14 | 2.22 | 0.15 | 3.02 | 0.54 | 2.05 | 0.22 | 4.13 | 1.15 | 2.94 | 0.49 | 2.41 | 0.41 | 2.39 | 0.27 |  |
| **PS(36:0)** | 58.70 | 14.78 | 18.17 | 7.00 | 46.63 | 20.06 | 12.75 | 2.72 | 41.16 | 8.80 | 87.77 | 40.67 | 25.86 | 9.72 | 23.83 | 9.28 |  |
| **PS(36:1)** | 43.45 | 2.08 | 46.79 | 3.05 | 65.17 | 6.86 | 51.31 | 5.16 | 93.59 | 30.87 | 47.07 | 4.19 | 63.63 | 7.31 | 50.31 | 5.53 |  |
| **PS(36:2)** | 0.58 | 0.04 | 0.57 | 0.04 | 0.87 | 0.13 | 0.54 | 0.08 | 1.15 | 0.34 | 0.84 | 0.11 | 0.73 | 0.10 | 0.69 | 0.10 |  |
| **PS(38:0)** | 30.53 | 1.39 | 35.90 | 2.35 | 47.43 | 7.11 | 40.06 | 3.49 | 64.88 | 20.26 | 42.26 | 5.25 | 44.31 | 4.21 | 35.95 | 2.61 |  |
| **PS(38:1)** | 37.99 | 1.83 | 43.54 | 2.24 | 44.92 | 4.45 | 46.99 | 4.23 | 69.81 | 18.93 | 41.52 | 3.41 | 48.90 | 3.79 | 44.32 | 4.60 |  |
| **PS(38:2)** | 30.47 | 2.45 | 38.49 | 3.36 | 27.35 | 3.04 | 37.39 | 4.55 | 43.08 | 8.13 | 34.50 | 3.34 | 28.08 | 2.13 | 21.94 | 3.44 |  |
| **PS(38:3)** | 16.96 | 0.80 | 19.77 | 1.67 | 32.96 | 4.81 | 22.30 | 2.62 | 30.52 | 6.83 | 18.87 | 1.63 | 24.85 | 2.39 | 23.24 | 1.79 |  |
| **PS(38:4)** | 1.35 | 0.08 | 1.47 | 0.12 | 2.38 | 0.38 | 1.27 | 0.14 | 2.02 | 0.35 | 1.73 | 0.23 | 1.47 | 0.14 | 1.80 | 0.21 |  |
| **PS(40:0)** | 2.98 | 0.18 | 3.48 | 0.23 | 5.49 | 0.76 | 3.98 | 0.31 | 7.54 | 2.51 | 3.48 | 0.33 | 3.91 | 0.59 | 5.75 | 1.27 |  |
| **PS(40:1)** | 14.16 | 1.33 | 16.80 | 2.48 | 15.61 | 1.56 | 19.00 | 3.45 | 22.65 | 6.26 | 23.24 | 3.62 | 24.35 | 2.78 | 13.04 | 1.81 |  |
| **PS(40:2)** | 15.58 | 0.98 | 18.86 | 1.37 | 17.56 | 2.22 | 18.09 | 1.72 | 25.21 | 6.10 | 18.89 | 1.79 | 17.49 | 1.56 | 14.80 | 1.35 |  |
| **PS(40:3)** | 16.78 | 0.73 | 20.65 | 1.43 | 24.94 | 3.07 | 23.27 | 2.53 | 27.72 | 5.05 | 18.63 | 1.36 | 21.67 | 1.79 | 21.43 | 1.53 |  |
| **PS(40:4)** | 8.01 | 0.38 | 9.19 | 0.64 | 13.84 | 2.22 | 9.99 | 0.67 | 13.10 | 3.15 | 9.68 | 0.95 | 10.15 | 0.97 | 12.37 | 1.45 |  |
| **PS(40:5)** | 5.60 | 0.28 | 7.02 | 0.57 | 9.80 | 1.16 | 8.27 | 2.09 | 10.00 | 2.55 | 6.26 | 0.56 | 8.97 | 1.23 | 7.80 | 0.73 |  |
| **PS(42:2)** | 0.81 | 0.07 | 1.00 | 0.07 | 0.98 | 0.13 | 0.83 | 0.13 | 1.35 | 0.36 | 1.11 | 0.09 | 1.18 | 0.18 | 0.65 | 0.08 |  |
| **PS(42:3)** | 7.15 | 0.41 | 9.35 | 0.76 | 10.13 | 1.00 | 10.80 | 1.06 | 12.69 | 2.13 | 9.14 | 0.95 | 8.78 | 0.70 | 8.81 | 0.61 |  |
| **PS(42:4)** | 12.14 | 0.48 | 14.44 | 0.79 | 16.06 | 1.82 | 14.94 | 1.33 | 17.84 | 3.24 | 13.91 | 1.02 | 11.76 | 0.90 | 16.25 | 1.26 |  |
| **PS(42:5)** | 2.49 | 0.16 | 2.90 | 0.29 | 5.29 | 0.73 | 3.14 | 0.49 | 4.20 | 1.19 | 2.97 | 0.40 | 4.32 | 0.42 | 4.20 | 0.65 |  |
| **PS(42:6)** | 0.34 | 0.03 | 0.45 | 0.08 | 0.64 | 0.15 | 0.58 | 0.10 | 0.37 | 0.05 | 0.46 | 0.07 | 0.41 | 0.08 | 0.61 | 0.08 |  |

Table 7. Average ± SEM Cer levels (µM) of sum composition species examined in all diagnostic groups with ε4 stratification. This data was QC normalized and semi-quantified using a Cer(d18:1/25:0) IS. The average number per group is shown for each diagnostic group and ε4 subgroups.

|  | **Ceramides (µM)** | | | | | | | | | | | | | | | | |
| --- | --- | --- | --- | --- | --- | --- | --- | --- | --- | --- | --- | --- | --- | --- | --- | --- | --- |
|  | **Control** | | | | **mTBI** | | | | **PTSD** | | | | **mTBI+PTSD** | | | | |
|  | n= 39 | | | | n= 12 | | | | n= 23 | | | | n= 5 | | | | |
| **APOE ε4** | **-** | | **+** | | **-** | | **+** | | **-** | | **+** | | **-** | | **+** | | |
|  | n= 27 | | n= 12 | | n= 8 | | n= 4 | | n= 15 | | n= 8 | | n= 3 | | n= 2 | | |
|  | **Mean** | **SEM** | **Mean** | **SEM** | **Mean** | **SEM** | **Mean** | **SEM** | **Mean** | **SEM** | **Mean** | **SEM** | **Mean** | **SEM** | **Mean** | **SEM** |  |
| **Cer(d36:1)** | 3.03 | 0.09 | 3.14 | 0.32 | 4.57 | 0.53 | 2.65 | 0.13 | 3.14 | 0.18 | 3.78 | 0.42 | 6.80 | 1.59 | 2.75 | 0.10 |  |
| **Cer(d38:1)** | 7.65 | 0.25 | 8.71 | 0.48 | 9.73 | 0.91 | 7.06 | 0.46 | 8.24 | 0.48 | 8.87 | 1.08 | 10.30 | 1.59 | 7.71 | 0.89 |  |
| **Cer(d40:1)** | 42.31 | 1.26 | 40.99 | 1.87 | 35.16 | 1.73 | 37.65 | 2.35 | 38.16 | 1.64 | 46.96 | 3.52 | 31.62 | 2.41 | 47.06 | 2.14 |  |
| **Cer(d41:2)** | 5.65 | 0.21 | 6.67 | 0.39 | 5.19 | 0.24 | 4.48 | 0.42 | 5.47 | 0.31 | 7.53 | 0.74 | 7.14 | 1.12 | 5.09 | 0.82 |  |
| **Cer(d42:0)** | 8.13 | 0.20 | 7.04 | 0.51 | 7.33 | 0.35 | 10.81 | 0.52 | 7.91 | 0.44 | 8.02 | 0.47 | 10.16 | 1.08 | 7.66 | 0.53 |  |
| **Cer(d42:1)** | 39.58 | 1.06 | 40.51 | 2.16 | 44.61 | 2.85 | 39.35 | 1.55 | 43.11 | 2.61 | 46.78 | 4.07 | 47.97 | 5.07 | 45.20 | 2.06 |  |
| **Cer(d42:2)** | 62.30 | 2.31 | 64.40 | 3.26 | 66.78 | 4.40 | 56.49 | 3.57 | 61.14 | 2.67 | 69.50 | 6.34 | 67.41 | 8.61 | 63.80 | 2.74 |  |
| **Cer(d43:2)** | 5.74 | 0.11 | 6.12 | 0.25 | 7.06 | 0.48 | 5.93 | 0.39 | 6.48 | 0.22 | 5.54 | 0.26 | 5.86 | 0.40 | 5.42 | 0.20 |  |

Table 8. Average ± SEM HexCer levels (µM) of sum composition species examined in all diagnostic groups with ε4 stratification. This data was QC normalized and semi-quantified using a 15:0-18:1(d7) PE IS. This IS was used as it was the closest eluting IS, however, it should be kept in mind that as this IS is of a different specie it’s extraction efficiency and response factor might have been different from that of HexCer species. The average number per group is also shown for each diagnostic group and ε4 subgroups.

|  | **Hexosylceramides (µM)** | | | | | | | | | | | | | | | |
| --- | --- | --- | --- | --- | --- | --- | --- | --- | --- | --- | --- | --- | --- | --- | --- | --- |
|  | **Control** | | | | **mTBI** | | | | **PTSD** | | | | **mTBI+PTSD** | | | |
|  | n= 34 | | | | n= 13 | | | | n= 26 | | | | n= 11 | | | |
| **APOE ε4** | **-** | | **+** | | **-** | | **+** | | **-** | | **+** | | **-** | | **+** | |
|  | n= 24 | | n= 10 | | n= 9 | | n= 4 | | n= 17 | | n= 9 | | n= 6 | | n= 5 | |
|  | **Mean** | **SEM** | **Mean** | **SEM** | **Mean** | **SEM** | **Mean** | **SEM** | **Mean** | **SEM** | **Mean** | **SEM** | **Mean** | **SEM** | **Mean** | **SEM** |
| **HexCer(d40:1)** | 4.31 | 0.17 | 3.70 | 0.24 | 3.80 | 0.30 | 4.65 | 0.51 | 3.95 | 0.15 | 3.77 | 0.22 | 4.23 | 0.46 | 4.89 | 0.39 |
| **HexCer(d41:1)** | 3.20 | 0.17 | 2.57 | 0.17 | 2.87 | 0.18 | 3.65 | 0.41 | 2.95 | 0.14 | 2.87 | 0.16 | 2.45 | 0.28 | 3.10 | 0.24 |
| **HexCer(d42:1)** | 7.72 | 0.29 | 6.79 | 0.38 | 6.52 | 0.48 | 7.99 | 0.92 | 7.30 | 0.30 | 6.40 | 0.48 | 6.19 | 0.72 | 8.78 | 0.82 |
| **HexCer(d42:2)** | 4.45 | 0.17 | 3.70 | 0.21 | 4.14 | 0.29 | 4.50 | 0.35 | 4.28 | 0.16 | 3.87 | 0.25 | 4.31 | 0.39 | 5.21 | 0.35 |
